# Multiexponential T2 relaxometry of benign and malignant adipocytic tumours

**DOI:** 10.1186/s41747-020-00175-0

**Published:** 2020-08-03

**Authors:** Katerina Nikiforaki, Georgios S. Ioannidis, Eleni Lagoudaki, Georgios H. Manikis, Eelco de Bree, Apostolos Karantanas, Thomas G. Maris, Kostas Marias

**Affiliations:** 1 Computational Bio-Medicine Laboratory (CBML), Institute of Computer Science (ICS), Foundation for Research and Technology - Hellas (FORTH), Nikolaou Plastira 100, Vassilika Vouton, GR-70013 Heraklion, Crete Greece; 2 Department of Radiology, School of Medicine, University of Crete, Heraklion, Greece; 3 Department of Pathology, University Hospital of Crete, Heraklion, Greece; 4 Department of Surgical Oncology, University Hospital of Crete, Heraklion, Greece; 5 Department of Medical Imaging, University Hospital, Heraklion, Greece; 6 Department of Medical Physics, University of Crete, Heraklion, Greece; 7 Department of Electrical & Computer Engineering, Hellenic Mediterranean University, Heraklion, Greece

**Keywords:** Lipoma, Liposarcoma, Magnetic resonance imaging, Neoplasms (adipose tissue), Subcutaneous fat

## Abstract

**Background:**

We investigated a recently proposed multiexponential (Mexp) fitting method applied to T2 relaxometry magnetic resonance imaging (MRI) data of benign and malignant adipocytic tumours and healthy subcutaneous fat. We studied the T2 distributions of the different tissue types and calculated statistical metrics to differentiate benign and malignant tumours.

**Methods:**

Twenty-four patients with primary benign and malignant adipocytic tumours prospectively underwent 1.5-T MRI with a single-slice T2 relaxometry (Carr-Purcell-Meiboom-Gill sequence, 25 echoes) prior to surgical excision and histopathological assessment. The proposed method adaptively chooses a monoexponential or biexponential model on a voxel basis based on the adjusted *R*^2^ goodness of fit criterion. Linear regression was applied on the statistical metrics derived from the T2 distributions for the classification.

**Results:**

Healthy subcutaneous fat and benign lipoma were better described by biexponential fitting with a monoexponential and biexponential prevalence of 0.0/100% and 0.2/99.8% respectively. Well-differentiated liposarcomas exhibit 17.6% monoexponential and 82.4% biexponential behaviour, while more aggressive liposarcomas show larger degree of monoexponential behaviour. The monoexponential/biexponential prevalence was 47.6/52.4% for myxoid tumours, 52.8/47.2% for poorly differentiated parts of dedifferentiated liposarcomas, and 24.9/75.1% pleomorphic liposarcomas. The percentage monoexponential or biexponential model prevalence per patient was the best classifier distinguishing between malignant and benign adipocytic tumours with a 0.81 sensitivity and a 1.00 specificity.

**Conclusions:**

Healthy adipose tissue and benign lipomas showed a pure biexponential behaviour with similar T2 distributions, while decreased adipocytic cell differentiation characterising aggressive neoplasms was associated with an increased rate of monoexponential decay curves, opening a perspective adipocytic tumour classification.

## Key points

- T2 relaxometry provides tissue/material specific information.
- The presented multiexponential method does not require assumptions on the number of T2 components.
- Healthy subcutaneous fat and benign lipomas showed pure biexponential T2 decay.
- Adipocytic tumours with decreased fat cell differentiation exhibit a relevant monoexponential contribution.

## Background

Tumours of adipocytic origin comprise the largest group of soft tissue tumours and can be both benign (lipomas) or malignant (liposarcomas). Liposarcomas, accounting for approximately 20% of all sarcomas, comprise a heterogeneous group consisting of tumours with different degrees of adipocytic differentiation, ranging from well-differentiated liposarcomas to tumours with intermediate adipocytic differentiation (myxoid liposarcomas) and poorly differentiated liposarcomas (pleomorphic, dedifferentiated, and round cell) [1]. Additionally, based on the National Cancer Institute and French Federation of Cancer Centers [2] scoring system on tumour differentiation, mitotic count and percentage of tumour necrosis, each liposarcoma is characterised by a histological grade of malignancy which ranges from G1 to G3 [3]. Histological type and grade are the most significant parameters in order to predict clinical behaviour, *i.e.,* rate of growth, possibility to metastasise, risk of recurrence, and survival rate.

Accuracy of the preoperative diagnosis, by means of identification of the correct adipocytic tumour type, as well as the definition of its histological malignancy grade is essential for patient management, including treatment planning [4]. In the case of malignant soft tissue tumours (sarcomas), wide excision of the tumour together with a rim of adjacent healthy structures is necessary to reduce the risk of local recurrence. Conversely, benign tumours are routinely treated by marginal resection [5]. Moreover, in sarcoma patients, preoperative non-surgical treatment might be indicated [4, 5]. Especially patients with myxoid liposarcoma seem to benefit considerably from preoperative radiotherapy [4]. Nowadays, accurate preoperative diagnosis is established only by tissue sampling, usually image-guided core needle biopsy. A precise noninvasive method for the differentiation between benign and malignant soft tissue tumours and the classification of their histological type is warranted.

From the imaging standpoint, soft tissue characterisation is derived from conventional as well as advanced MRI protocols. Taking into account that the radiological appearance of soft tissue tumours on conventional imaging is commonly not conclusive for histological type and tissue grading, biomarkers from advanced protocols can be deployed for noninvasive preoperative assessment [6, 7]. One of the best established biomarkers for tissue characterisation is transverse magnetisation (T2) relaxometry, utilised for a large variety of clinical [6–8] and non-clinical applications [9–11] as it provides information from the inner structure of the imaging object and is also independent of reader, pulse sequence, coil, or imaging parameters. Notably, the T2 decay is affected by the tissue free water content, fraction of water bound to molecules and macromolecules, local tissue temperature, tissue fat content, presence of paramagnetic particles, and pH value [9]. Consequently, an appropriate analysis may reveal information from the inner structure of tissue at a molecular scale.

Many recent studies utilise more than one exponential components to provide greater specificity to the water proton transverse relaxation. Multicomponent T2 analysis studies the pattern of relaxation in complex systems described by distinct types of interacting proton groups: solid macromolecules (fast T2 decay), water protons bound to lipids and proteins (intermediate T2 decay), and free water protons (slow T2 decay) [12–14]. Moreover, lipid protons contribute to the signal in fat-containing tissues with the expected T2 pattern from fat globules being a bimodal distribution with intermediate T2 values [15].

Our aim was fat characterisation based on a multiexponential (Mexp) framework assuming that decay components may be one or two which is deployed to characterise tissue of different fat cell differentiation, *i.e.,* histological resemblance to normal fat cells. This method has been previously tested on a phantom with samples of aqueous, fatty, and mixed composition [16]. To the best of our knowledge, it is the first voxel-based multi-compartment description of T2 relaxation properties of human adipocytic tumours.

## Methods

### Imaging protocol

All MRI exams were performed on a 1.5-T scanner (Vision/Sonata hybrid System, Siemens, Erlangen, Germany). Conventional T1-weighted as well as dual-echo proton density- and T2-weighted turbo spin-echo sequences offered complete coverage of the body region in question and were used for localising the quantitative study. The field of view and the choice of the coil depended on the site of the tumour and were determined on the basis of the potential highest signal-to-noise ratio and adequate spatial coverage. Two possible schemes were used for the field of view (FOV), either 200 × 200 mm or 400 × 400 mm as masses differed substantially in their size and location. The T2 quantitative MRI protocol consisted of two-dimensional (2D) single-slice multi-echo spin-echo based on a 2D multi-echo spin-echo Carr-Purcell-Meiboom-Gill sequence with alternating 180° radiofrequency pulses under the phase-alternating-phase-shift scheme [17]. The final result was a single-slice, proton density-to-T2-weighted sequence with repetition time equal to 2,500 ms, 25 equidistant spin echoes, with the first echo time (TE) at 13.4 ms, echo spacing 13.4 ms, and last TE at 335 ms. Slice thickness was 5 mm and a rectangular reconstruction matrix (384 × 320 pixels) was chosen. A selective refocusing radiofrequency pulse scheme was utilised for elimination of stimulated echoes [18].

### Patient population and histopathology

The imaging protocol was submitted and approved by the local ethics committee. All patients signed an informed consent for the use of clinical and imaging data for research purposes. Patients with primary lipomatous tumours who underwent MRI examination under a prospectively set protocol from July 2017 to February 2019 prior to the planned surgical excision were included in the study. Patients with recurrent or residual tumours and those submitted to preoperative treatment were excluded. Subsequently, we excluded from analysis one patient due to severe motion artifacts and two patients that refused to perform the examination because of claustrophobia. A total of 24 patients with primary lipomatous tumours of the lower limb (*n* = 11), the upper limb (*n* = 7), and the retroperitoneal area (*n* = 6) were included.

The surgeon marked the specimen with sutures or surgical staples at predefined points in order to enable the actual three-dimensional orientation of the specimen in relation with the patient’s body and to warrant the implementation of sections of the tumour along a true axial plane. For each patient, at least three slices (at the top and bottom end and a slice in the middle of the tumour) were analysed for differentiation, cell type, cellular atypia, cellularity, mitotic activity, vascularity, and presence of necrosis. Histopathologic analysis revealed six lipomas, four well-differentiated liposarcomas, three myxoid liposarcomas, four pleomorphic liposarcomas, and seven dedifferentiated liposarcomas. Within tumours of increased heterogeneity, the pathologist guided the radiologist to identify regions indicative of myxoid or fibrous composition as well as areas of well differentiated or poorly differentiated tissue on the MRI slices.

### Data preprocessing

In the multi-echo spin-echo phase-alternating-phase-shift sequence, the first echo signal is not accurate because of B_1_ field inhomogeneity [18, 19] and is usually either extrapolated or ignored. In this study, we did not correct for the first echo as extrapolation process requires the choice of a proper model and this would in turn introduce bias to the next step of monoexponential or biexponential fitting model selection. The first TE was therefore the second acquired at 26.8 ms from the original sequence.

### T2 relaxometry (Mexp method)

All numerical calculations concerning Mexp T2 relaxometry were implemented in Python 3.5 (www.python.org) apart from the inverse Laplace transform method which was implemented in Matlab 2015a (Mathworks, Natick, MA, USA). The graphical user interface and result visualisation were accomplished by the use of PyQt4 and PyQtGraph (www.pyqtgraph.org) libraries respectively. The mathematical framework and technical details for the T2 relaxometry are presented below.

Supposing a signal *S*(TE_k_), measured at echo times TE_k_ (*k* = 1, 2 ,…, *K*), the decay of the transverse magnetisation can be represented as the sum of up to *N* exponential decays as shown in (1).

1 $$ S\left({\mathrm{T}\mathrm{E}}_{\mathrm{k}}\right)-e\left({\mathrm{T}\mathrm{E}}_{\mathrm{k}}\right)=\sum \limits_{i=i}^N{A}_{\mathrm{i}}\exp \left(-\frac{{\mathrm{T}\mathrm{E}}_{\mathrm{k}}}{\mathrm{T}{2}_{\mathrm{i}}}\right),\kern1em N=1,2 $$

*e*(TE_k_) was considered to be the vector containing the mean background noise for every TE which was subtracted from the signal. Our analysis was based on searching a maximum of two components since the protocol did not permit to search for very short T2 values since the first TE was at 26.8 ms. Every voxel curve was fitted twice in Eq. (1) for all *N* (*N* = 1,2) meaning both monoexponential and biexponential fit as a preparatory step by using non-linear least squares. The number of exponentials per voxel fit was determined by the highest ${\overline{R}}^2$ described in the “Goodness of fit” section and will be referred to as R2 criterion hereinafter. The search space for each unknown variable was set as *A*_i_, (*i* = 1, 2) in [0, 2,000], *T*2_1_  ∈  [27, 120] ms and *T*2_2_  ∈  [120.1, 2,500] ms. For the case of *N* = 1 the T2_1_ was set to [27, 2,500] ms. The intervals for T2_i_ were determined firstly by sequence limitations to detect very short T2 solid or tightly bound components and secondly by reported T2 values in bibliography [20–23] for weakly bound and free water components. More extensive information on the Mexp method is presented in [16].

### Goodness of fit

Having an analytical form of the model fitted to the data, the adjusted *R* squared $\left({\overline{R}}^2\right)$ can be computed in order to acquire information about the goodness of fit. ${\overline{R}}^2$ was considered more suitable for this study than *R*^2^ since it takes into account the number of data points (*K*), the number of the explanatory variables (*m*) of the model function and the residuals between the model function (*G*_*i*_) and the data (*y*_*i*_) as can be seen in Eqs. (2) and (3). Index *i* stands for the number of the measured data points.

2 $$ {\overline{R}}^2=1-\left(1-{R}^2\right)\frac{K-1}{K-m-1} $$

3 $$ {R}^2=1-\frac{\sum_{i=1}^k{\left({G}_i-\overline{y}\right)}^2}{\sum_{i=1}^k{\left({y}_i-\overline{y}\ \right)}^2} $$

**Fig. 1:**
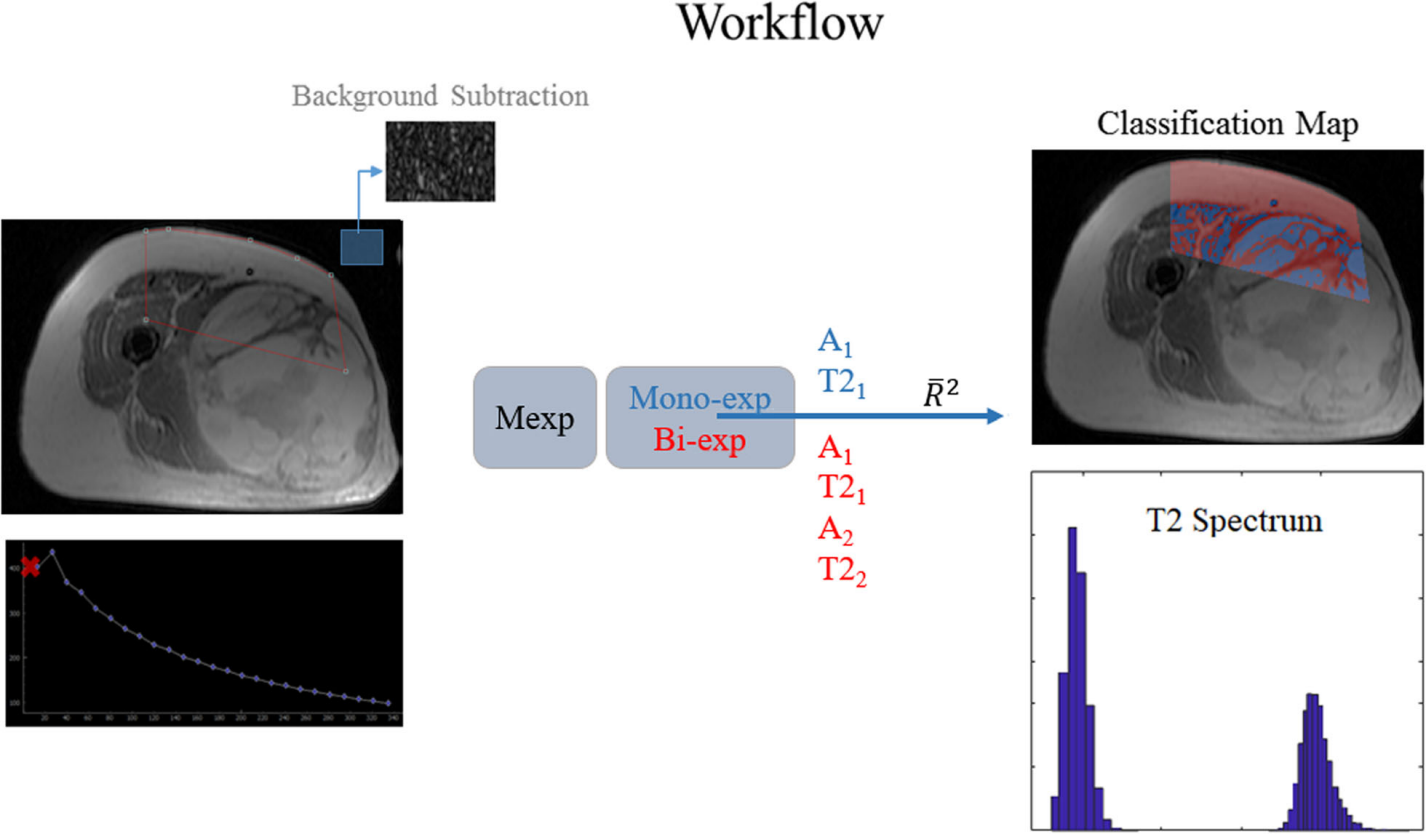
Study workflow

A graphical representation of the workflow used in this study is shown in Fig. 1.

### Voxelwise analysis

For each patient, an expert radiologist with more than 12 years of experience in body MRI annotated the tumour region for the patient cohort. Voxelwise parametric maps were produced by the Mexp method for all patients. All voxels assigned as tumour from all patients were used to constitute five tissue categories (benign lipoma, well-differentiated liposarcoma, myxoid liposarcoma, poorly differentiated liposarcoma, pleomorphic liposarcoma) according to the histopathologic diagnosis. The radiologist also annotated healthy subcutaneous fat ROIs for each patient to be used for reference purposes.

### Classification analysis

Initially, a stratified 5-fold cross validation technique was applied to the dataset in order to prevent sample selection bias. From the Scikit-learn python library [24], logistic regression was used for the discrimination between the two classes, *i.e.,* malignant and benign tissue types, and for the calculation of sensitivity and specificity metrics. More specifically, benign tissue category contained the lipoma and health subcutaneous fat, while malignant category included ROIs from well-differentiated liposarcomas, myxoid liposarcomas, pleomorphic liposarcomas, and dedifferentiated liposarcomas. The statistical metrics used for the analysis were as follows: mean value, standard deviation (SD), and the 90th percentile for each one of % model prevalence, T2_1_ and T2_2_ values.

## Results

**Fig. 2:**
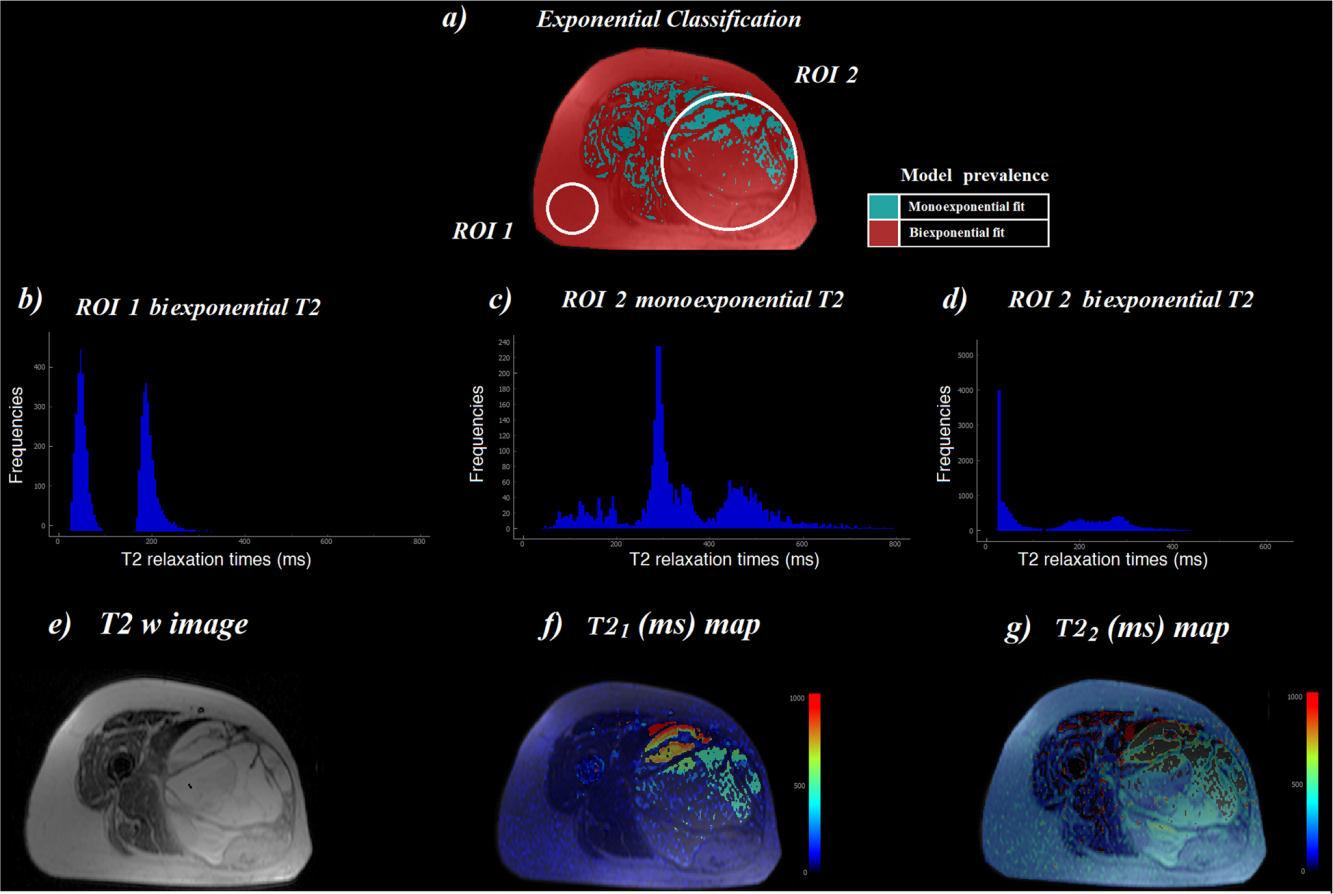
**a** Whole slice Mexp analysis for a lower limb sarcoma where ROI_1 is indicative for healthy adipose tissue area and ROI_2 corresponds to a malignant region. **b**–**d** Mexp-derived spectra for ROI_1 and ROI_2. **e** T2-weighted images. **f**, **g** Whole slice T2_i_ parametric maps

For each category, the resulting Mexp parameters (relative amplitudes *A*_i_ and corresponding T2_i_ values) as well as the relative contribution of either monoexponential or biexponential fitting are presented in Table 1. A graphical overview of the Mexp results, *i.e.,* derived parametric maps and spectra, for healthy (ROI_1) and malignant tissue (ROI_2) are presented in Fig. 2. In detail, (a) shows voxel-based classification with respect to the optimal number of exponentials based on the R2 criterion, (b) T2_1_ for monoexponentially fitted voxels, (c, d) T2_1_ and T2_2_ for biexponentially fitted voxels, respectively, (e) anatomical T2-weighted image of the analysed slice, and (f, g) T2_1_ (short) and T2_2_ (long) parametric maps for the whole slice, respectively. All delineated ROIs were similarly analysed and results from each patient were grouped according to tissue histologic subtype.

**Table 1:** Multiexponential T2 analysis

Tissue type	Monoexponential/biexponential prevalence (%)	*A*_1_ ± SD (a.u.)	T2_1_ ± SD (ms)	*A*_2_ ± SD (a.u.)	T2_2_ ± SD (ms)
Lipoma	0.3% monoexponential	–	–	–	–
	99.7% biexponential	319 ± 183	41 ± 13	630 ± 195	205 ± 64
Well-differentiated liposarcoma	17.7% monoexponential	205 ± 95	322 ± 139	–	–
	82.3% biexponential	279 ± 160	42 ± 19	757 ± 260	220 ± 112
Myxoid liposarcoma	47.8% monoexponential	910 ± 134	439 ± 79	–	–
	52.2% biexponential	237 ± 129	60 ± 37	793 ± 190	469 ± 148
Poorly differentiated liposarcoma	53.0% monoexponential	469 ± 237	152 ± 102	–	–
	47.0% biexponential	242 ± 156	48 ± 25	525 ± 378	223 ± 121
Pleomorphic liposarcoma	25% monoexponential	583 ± 99	441 ± 154	–	–
	75% biexponential	285 ± 95	38 ± 19	678 ± 109	263 ± 153
Adipose tissue	100% biexponential	504 ± 69	45 ± 13	751 ± 147	191 ± 21

*a.u.* Arbitrary units, *A*_*1*_ Relative amplitude of the short T2 component, *A*_*2*_ Relative amplitude of the long T2 component, *SD* Standard deviation, *T2*_*1*_ Short relaxation time, *T2*_*2*_ Long relaxation time

**Fig. 3:**
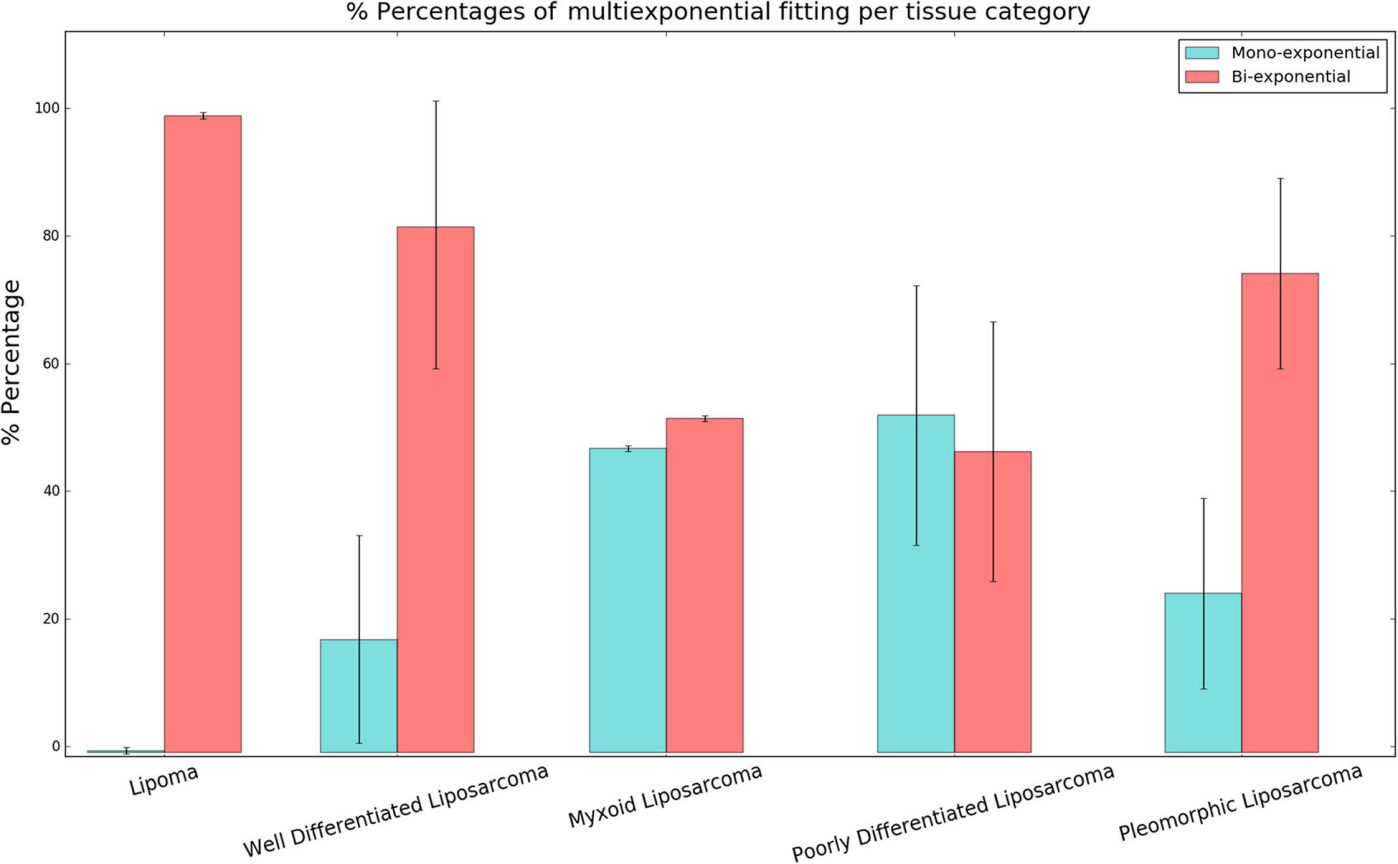
Relative contribution of monoexponential and biexponential behaviour for each tumour subtype

**Fig. 4:**
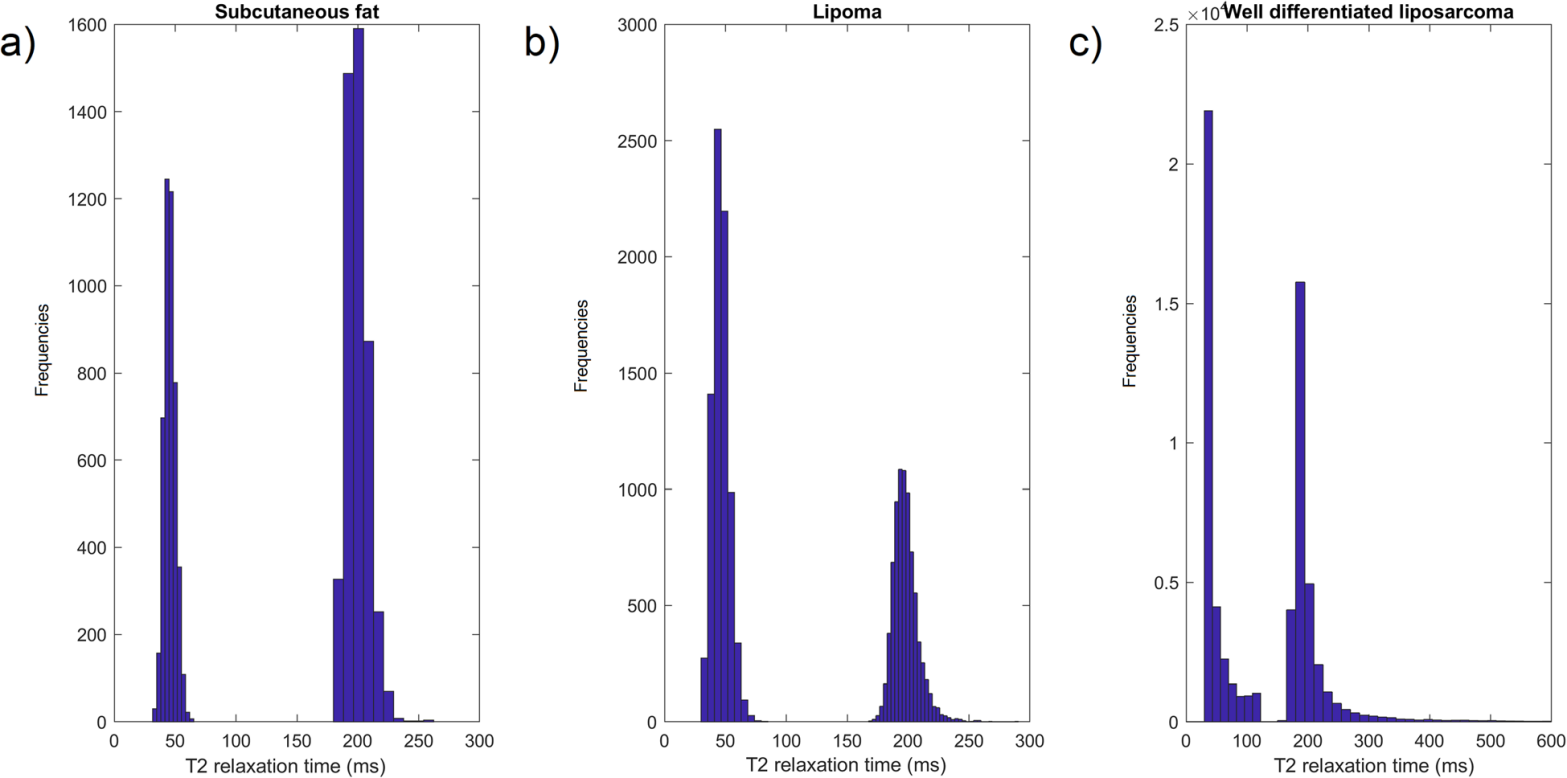
Derived Mexp spectra from purely biexponential tissue samples for subcutaneous fat (**a**), lipoma (**b**), and well-differentiated liposarcoma (**c**)

**Fig. 5:**
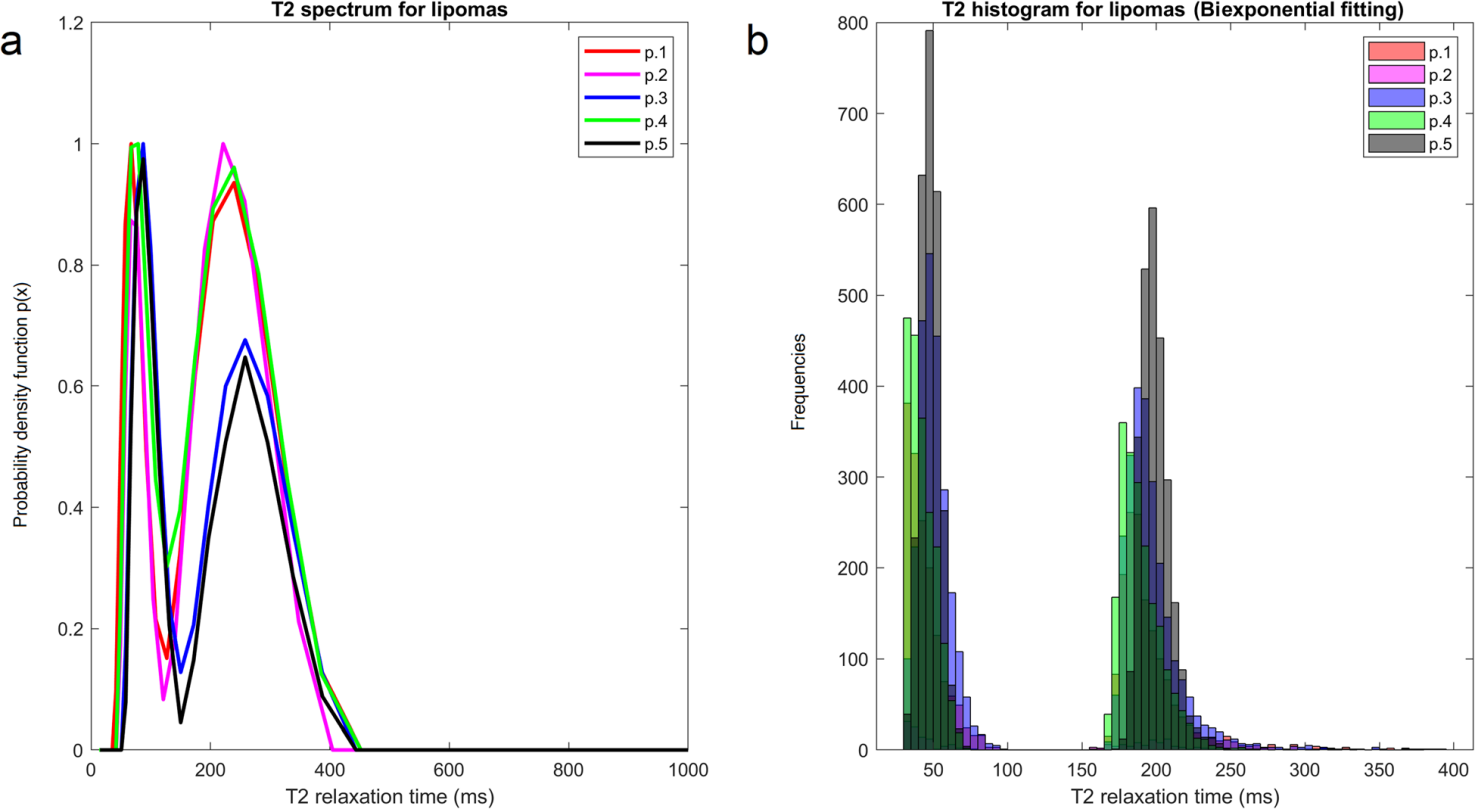
Per-patient analysis of T2 relaxometry data with inverse Laplace method (**a**) and Mexp method (**b**) for five lipoma patients

To gain a rough insight into tissue T2 decay pattern, firstly the relative monoexponential or biexponential contributions are graphically presented in Fig. 3. For tissue samples exhibiting pure biexponential behaviour (healthy subcutaneous fat, benign lipoma, well-differentiated liposarcoma) Mexp spectra are shown in Fig. 4. Moreover, for all patients with a lipoma diagnosis, we additionally performed inverse Laplace transform, in order to examine similarities in the results derived by Mexp method to the most widely used one. This additional feature (Fig. 5) also presents similarity of spectra among patients for both methods.

**Fig. 6:**
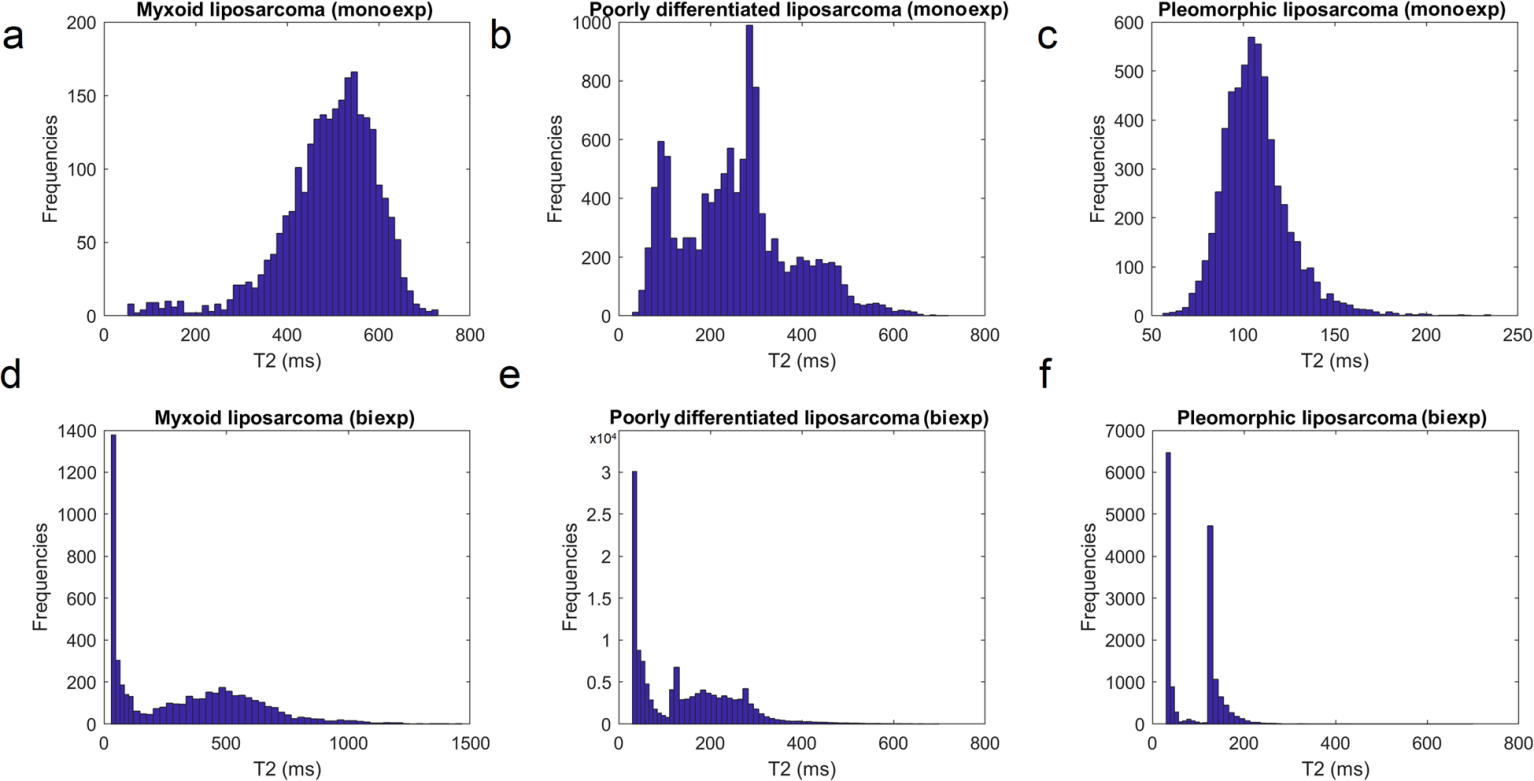
Derived spectra from mixed monoexponential and biexponential tissue samples for myxoid liposarcoma monoexponential spectrum (**a**), poorly differentiated liposarcoma monoexponential spectrum (**b**), pleomorphic liposarcoma monoexponential spectrum (**c**), myxoid liposarcoma biexponential spectrum (**d**), poorly differentiated liposarcoma biexponential spectrum (**e**), and pleomorphic liposarcoma biexponential spectrum (**f**). *exp* Exponential

**Fig. 7:**
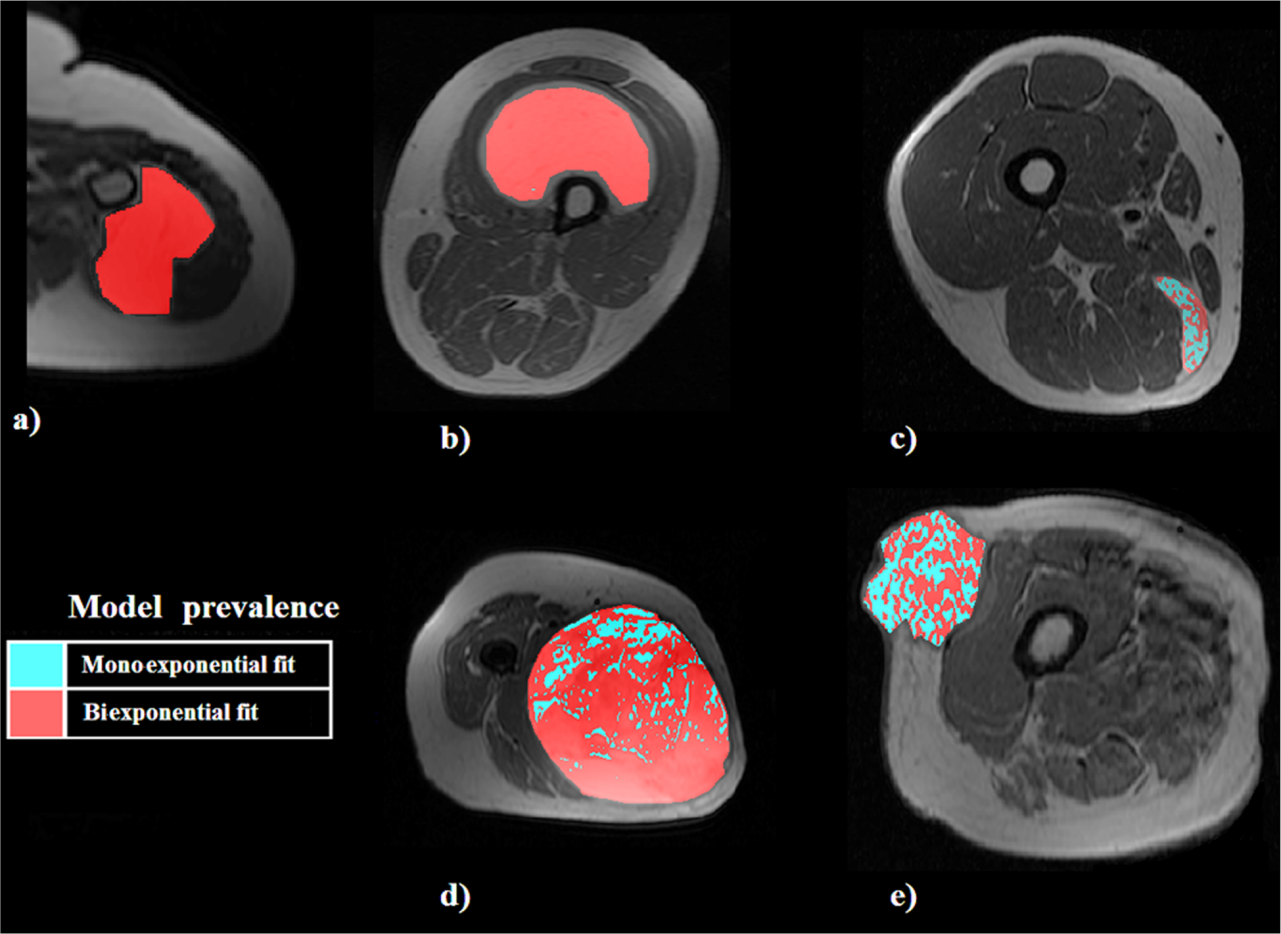
Voxelwise model classification from Mexp for five patients with different histopathologically proven lipomatous neoplasms: benign lipoma (**a**), well-differentiated liposarcoma (**b**), myxoid liposarcoma (**c**), poorly differentiated liposarcoma (**d**), pleomorphic liposarcoma (**e**)

For tissues of higher grade of malignancy, (myxoid liposarcoma, poorly differentiated liposarcoma, pleomorphic liposarcoma) the Mexp-derived spectra for both short and long T2 components are shown in Fig. 6, while an indicative slice for each tissue category presenting monoexponential and biexponential dominance is shown in Fig. 7. Furthermore, the classification results for benign and malignant adipose tissue categories are summarised in Table 2, presenting the most discriminant metrics of the classification.

**Table 2:** Classification analysis

Statistical metrics	Sensitivity	Specificity
Mean (% model prevalence)	0.81	1.00
Mean (T2_1_)	0.85	0.77
Standard deviation (T2_1_)	0.89	0.88
90th percentile of T2_1_	0.91	0.92
Mean (T2_2_)	0.87	0.81
Standard deviation (T2_2_)	0.89	0.73
90th percentile of T2_2_	0.85	0.88

*T2*_*1*_ Short relaxation time, *T2*_*2*_ Long relaxation time

## Discussion

On the historical background, the driving force for expanding multiexponential T2 methodology armamentarium has been the study of myelin in the brain and muscle. Concerning the former, three distinct water compartments have been identified, assigned to motion-restricted water particles between the lipid layers of the myelin sheath (10 ms < T2 < 40 ms), more freely moving water in the intracellular and extracellular spaces (80 ms < T2 < 100 ms) and cerebrospinal fluid (T2 > 2,000 ms) [20, 21, 25]. For the latter, studies on muscle tissue have identified up to four water compartments with T2 values ranging from < 5 to T2 > 400 ms, assigned to the following: (1) extracellular exponential extracellular component (T2 > 400 ms), (2) intermyofibrillar component (T2≈150 ms) (3) myofibrillar component (30 ms < T2 < 60 ms), and (4) a rapid component residing at the vicinity of the macromolecules (T2 < 30 ms) [22, 26].

In the specific domain of lipid or connective tissue, T2 relaxometry has been the subject of a limited number of publications [23, 27]. However, reported results from magnetic resonance spectroscopy (MRS) have shown evidence that the study of fat content allows for adipose tissue classification on the basis of variable abundance of distinct fat moieties with each one having a different T2 value in benign lipomatous tissue and liposarcomas [28, 29]. Adipose tissue differentiation based on MRS has shown a marked fall in visible triglyceride levels associated with a significant increase in phosphatidylcholine levels, with the latter serving as a measure of cell membrane turnover and cell division. Low-grade liposarcoma was found to have similar triglyceride content to normal fat (25). Notwithstanding the significance of these results, *in vivo* MRS is not readily available and moreover has poor spatial resolution and has high software, timing, and shimming demands, as opposed to T2 relaxometry [30].

MRI signal in clinical sequences comes from protons in water or lipids that mainly occur as fatty droplets within the cells of adipose tissue. As mentioned earlier, water protons exhibit different T2 values, depending on molecular motion, while protons from lipids exhibit more complex T2 distributions (bimodal) where each mode cannot be directly attributed to a specific molecular group of the fatty acid chain. Longer T2 are conjectured to originate from methyl-H in mobile triglycerides from cytoplasmic lipid droplets, most abundant in highly differentiated liposarcomas, while, on the other hand, shorter T2 components come from rotationally hindered and perhaps intracellularly bound protons of pleomorphic or dedifferentiated liposarcomas. Myxoid neoplasms exhibit higher water content and low triglyceride and increased levels of phospholipids [28].

In our study, the use of different coils or FOVs was necessary since tumour sites were different across the patient cohort. Smaller FOV (200 × 200 mm) was used for upper or lower limb imaging, while abdomen imaging was performed with a larger FOV (400 × 400 mm). The choice of FOV and coil was made in order to ensure adequate signal-to-noise ratio, full lesion coverage, and mitigation of artifacts.

In this study, we described tissue in terms of monoexponential or biexponential T2 relaxation per voxel and resulting T2 values as measures indirectly indicative of tissue composition. Tissue heterogeneity was also evident as heterogeneous pattern of relaxation. Adipose tissue and benign lipomas exhibited pure biexponential behaviour, which is in accordance with phantom-based studies with tissue mimicking materials, such as corn oil [31]. This finding of pure biexponential was consistent among all examined samples, explaining the small standard deviation in Fig. 3. The purpose of performing a per-patient analysis for patients diagnosed with lipoma was to examine the consistency of the results among patients and also to compare the Mexp-derived results with the widely used inverse Laplace transform method, which was used as ground truth. The advantage of Mexp compared to inverse Laplace method is the ability for voxelwise parametric mapping, whereas the latter method provides a T2 distribution over the entirety of the examined volume of interest. Thus, it lacks the ability of efficient examination of local tissue heterogeneity.

Similarity of well-differentiated fat to normal fat and benign lipoma as observed at histopathology is also reflected in the decay patterns and resulting hardly separable distributions. All three entities show pure biexponential behaviour and similar T2 values. Myxoid neoplasms exhibited higher T2 values and significant contribution from the monoexponential compartment which can be attributed to the myxoid pools of the neoplasm. The consistency in the percentages of monoexponential or biexponential decay patterns in the limited number of the examined cases resulted in narrow standard deviations as illustrated by the error bars in Fig. 3.

Concerning tumours of high malignancy and lower adipose cell differentiation, a very interesting initial finding is that, despite the similarity in signal intensity as shown in conventional T2-weighted images (Fig. 2e), there was an obvious difference in the relaxation pattern of healthy and poorly differentiated adipose tissue (Fig. 2a) and also in the measured T2 relaxation constant (Fig. 2 f and g). However, more extensive analysis needs to be done in order to support this initial finding. Lastly, we separately examined and observed shortened T2 times and monoexponential behaviour in the areas of necrosis identified by histopathology, potentially as a result of macromolecular compounds from the cells released into the extracellular space and binding to free water molecules [32].

Additionally, the first (effective) TE is limiting the minimum T2 value feasible to be detected by the proposed method and thus one cannot exclude a third compartment in the low T2 range that cannot be detected with the adopted sequence parameters. A very short T2 component could be attributed to water protons motionally restricted, probably strongly bound to macromolecules or physically constrained within cell structures. The minimum bound for T2 estimation in this study was set at 27 ms, corresponding to the first effective TE of the echo train.

In this study, the classification was performed between benign and malignant adipose tissue types, presenting a limited clinical impact. The most discriminant metrics between the two categories were the mean (% model prevalence) and the 90th percentile of T2_1_ with sensitivity/specificity of 0.81/1.0 and 0.91/0.92, respectively. In the frame of a larger patient cohort, a classification based on T2 distribution metrics could be performed among the different tissue types (lipoma, well-differentiated liposarcoma, myxoid liposarcoma, pleomorphic liposarcoma, and dedifferentiated liposarcoma). Such a calculation will present increased clinical interest since it could help in the differential diagnosis between clinical entities with overlapping radiological characteristics.

Our limited sample size does not allow for classification of the various types of liposarcomas. However, we showed the complex pattern of relaxation among different tissue types that can be sensitive to the chemical and physical environment surrounding water hydrogen protons. The pure biexponential behaviour characterising healthy adipose tissue and benign lipoma differs for the malignant tissues of lipomatous origin, where a monoexponentially decaying component contributes to the signal time intensity curve of Carr-Purcell-Meiboom-Gill phase-alternating-phase-shift scheme T2 relaxometry data.

In conclusion, we presented the results of a Mexp T2 relaxometry method assuming that decay components may be either one or two for the analysis of adipocytic tumours. The advantage of this method is the ability to provide voxelwise valuable tissue-specific information which stems from a microscopic tissue scale. In future large-scale studies, model percentages and individual T2_i_ components may serve as biomarkers for tumour classification, *i.e.,* an adjunct to conventional radiological assessment of the complex tissue microenvironment.

## Data Availability

There is no consent to share the patient data for further analyses.

## Abbreviations

$$ {\overline{R}}^2 $$: Adjusted *R*^2^
2D: Two-dimensional
FOV: Field of view
Mexp: Multiexponential
MRI: Magnetic resonance imaging
MRS: Magnetic resonance spectroscopy
ROI: Region of interest
SD: Standard deviation
TE: Echo time
